# Over‐Transfusion and Unnecessary Transfusion Following Post‐Partum Haemorrhage at Te Toka Tumai Auckland Hospital

**DOI:** 10.1111/ajo.70066

**Published:** 2025-07-31

**Authors:** J. Stefanus Grobler, Lynn C. Sadler, John Thompson, Matthew Drake, Beatrice Treadwell, Jenny McDougall, Meghan G. Hill

**Affiliations:** ^1^ University of Auckland Auckland New Zealand; ^2^ Te Toka Tumai Auckland Te Whatu Ora Auckland New Zealand

**Keywords:** blood transfusion, haemorrhage, parturition, postoperative haemorrhage, transfusion

## Abstract

**Background:**

Blood transfusion is an important treatment for obstetric haemorrhage. Transfusion also engenders significant short and long‐term risks. Ensuring blood products are only given when necessary is a priority in improving outcomes.

**Aims:**

To describe the population transfused at a single unit in New Zealand and identify the proportion of patients over and unnecessarily transfused via adjustment of haemoglobin per unit of blood given. To assess whether the rate of inappropriate transfusion was modified by demographic and treatment characteristics.

**Materials and Methods:**

A retrospective cohort study inclusive of all people who gave birth from 20 weeks between 2018 and 2021 at one hospital was assembled. People who were administered red blood cell‐containing products were identified. The pre‐discharge haemoglobin was adjusted per unit of blood given with patients being considered over or unnecessarily transfused at a pre‐discharge haemoglobin of ≥ 90 mg/dL.

**Results:**

The transfused population comprised 694/25 915 pregnancies (2.7% of the cohort). Appropriate transfusion (pre‐discharge haemoglobin < 90) occurred in 332/694 (47.8%) people. There were 325 (46.8%) patients who were over‐ or unnecessarily transfused. There was no difference in appropriateness of transfusion for any ethnicity compared to Māori, our referent group. Over‐transfusion rates did not differ and were high in both acute (53%) and non‐acute (45%) settings.

**Conclusion:**

The rate of transfusion for obstetric haemorrhage was 2.7% in our study population. Approximately half of people receiving blood received either too many units or did not require a transfusion.

## 1 Introduction

Post‐partum haemorrhage (PPH) is a common, life‐threatening complication of both vaginal and caesarean births. PPH is defined as significant maternal blood loss that occurs during or after the birth of a baby, which may be accompanied by signs or symptoms of hypovolaemia. Uterine blood flow at term is approximately 600 mL/min, and catastrophic haemorrhage can occur rapidly with injury or atony; the subsequent severe anaemia, disseminated intravascular coagulation, multisystem organ failure and maternal cardiac arrest can result in death within minutes of the haemorrhage onset [1]. PPH is the most common preventable cause of maternal mortality globally [2]. In Aotearoa New Zealand, obstetric haemorrhage is responsible for 2.9% of maternal deaths [2, 3].

Transfusion of blood products has a core role in treating PPH, both in managing the underlying anaemia associated with blood loss and halting ongoing haemorrhage (through transfusion of clotting factors). Guidelines for transfusion during PPH vary regionally in NZ even though national guidelines exist [4, 5, 6]. Notably, guidelines at Te Toka Tumai Auckland Hospital suggest that transfusion, or preparation for transfusion of blood products, should begin at 1500 mL blood loss [4]. However, Te Toka Tumai Auckland Hospital annual clinical reports indicate that about 60% of people with a blood loss > 1500 mL do not receive transfusion [7]. These rates of transfusion may be appropriate as administration of blood products is associated with an increased risk of all‐cause morbidity alongside transfusion‐specific complications [8, 9]. Furthermore, transfusions are a costly intervention compared to conservative alternatives, and their overuse represents poor utilisation of healthcare resources.

The non‐obstetric literature has suggested that over‐transfusion is common even in hospital settings with robust protocols [10]. Findings in obstetric populations vary with over‐transfusion ranging from 26% to 52% [11, 12]. These obstetric studies did not identify the rate of unnecessary transfusions in their populations.

The aims of this study are to describe the population of transfused patients at Te Toka Tumai Auckland Hospital; to identify the rate of over‐transfusion and unnecessary transfusion among transfused patients; and to compare those that were appropriately transfused with those over‐transfused and unnecessarily transfused by demographic characteristics and by timing of transfusion.

## 2 Methods

This retrospective cohort study included all patients who birthed at Te Toka Tumai Auckland Hospital between 1st January 2018 and 31st December 2021. The transfusion group included patients who received packed red cell or whole blood transfusion within 6 weeks of birthing for an obstetric indication. All included patients birthed from 20 weeks gestation.

Ethical approval was granted by the New Zealand Health and Disability Ethics Committee (HDEC), (2024 EXP 19647), and site approval by Te Toka Tumai Auckland research office (A+10009).

### 2.1 Inclusion and Exclusion Criteria

Post‐partum transfusion was defined as receipt of blood products containing red blood cells (BPCRBC), that is, packed red cells and whole blood, within 6 weeks of birth for an obstetric indication. Obstetric indications were ones where the main source of bleeding, which resulted in transfusion, was an expected part of or a complication of the birthing process. Transfusions for non‐obstetric indications were excluded after consultation with the research team.

Patients who received only cell‐saver autologous blood transfusions or component blood products that did not contain red blood cells (fresh frozen plasma, albumin, platelets or cryoprecipitate) were excluded.

In the case of multiple pregnancies, the mother is represented once, along with the baby with the most invasive birth method (prioritised as emergency caesarean > elective caesarean > instrumental birth > breech vaginal birth > cephalic vaginal birth). If birth modality for all babies was the same, the multiple with the largest birth weight was retained. Patients could be included in the sample more than once if they had more than one pregnancy and subsequent birth during the study period.

### 2.2 Data Collection and Variable Derivation

Routinely collected data, entered by clinical and administrative staff during pregnancy, were provided to the researchers. All records of patients with a reported transfusion, at any postnatal admission within 6 weeks of birth, were manually reviewed and transfusion related information collected. The number of units of blood transfused was collected from blood tracing stickers on blood product administration records. Patients who did not have a completed blood administration record who had evidence of transfusion in their clinical notes were discussed by the research team to determine if they met inclusion criteria. Maternity clinical records were checked for all patients with an estimated post‐partum blood loss of greater than 1500 mL who were not recorded as transfused, and these patients were included if they met inclusion criteria.

Pre‐discharge haemoglobin (pdHb) was defined as the last recorded haemoglobin measurement following transfusion and prior to discharge from the admission in which the patient was transfused. The primary means of measurement was blood samples sent to the hospital pathology laboratory, although point‐of‐care testing modalities were sometimes used in theatre or in the post‐anaesthetic care unit. These have been found to be as reliable as standard laboratory testing [13].

In theatre, blood loss is estimated using the sum of the volume retrieved via suction, caught within the surgical drapes and weighed on swabs, and subtracting the volume of liquor and wash used. Outside of theatre, blood loss is typically estimated by comparing soiled linens or hygiene products with standardised charts or by weighing these items to determine the weight of absorbed fluids.

Timing of transfusion was divided into two groups based on the perceived urgency of transfusion: Acute (including the delivery unit, emergency department, in the women's health admissions unit or the postoperative acute care setting) and non‐acute (including general and intensive care wards).

‘Maternal deprivation quintile’ combines ‘deprivation deciles’, quintile five being the most deprived group, and is derived from a census area‐based measure of socioeconomic deprivation [14]. ‘Maternal prioritised ethnicity’ reports prioritised ethnicity categories recommended by The Ministry of Health, and used for New Zealand obstetric research [15].

### 2.3 Calculations of Over‐Transfusion and Unnecessary Transfusion

The cut‐off for over‐transfusion was determined to be a pdHb of 90 mg/L as previously used in obstetric research [11]. Patients with a pdHb of less than 90 g/L were considered appropriately transfused. Patients with a pdHb of greater than or equal to 90 g/L were considered over‐transfused. It has been demonstrated that a unit of BPCRBC increases the haemoglobin of the average person by 10 g/L [16, 17, 18]. This assumption was used to correct the pdHb of over‐transfused patients based on the number of units transfused.

The over‐transfused group was divided into two subgroups. After the adjustment of 10 g/L per unit of blood, those with a corrected pdHb of less than 90 g/L were considered over‐transfused but likely required at least one unit of BPCRBC. Patients with a corrected pdHb of greater than or equal to 90 g/L were considered to have not required any transfusion.

### 2.4 Accuracy Checks

BMI data was checked for all patients with a BMI greater than 60 kg/m^2^ or less than 15 kg/m^2^. Height was manually checked if recorded to be less than 135 cm and weight was manually checked if recorded to be greater than 200 kg.

### 2.5 Data Analysis

Data was collected into a Microsoft Excel file protected in a SharePoint digital workspace. Statistical analysis was completed using STATA 18.0 [19]. It consisted of descriptive statistics and comparisons using chi‐squared tests for frequency data, student t‐tests for parametric and rank sum tests for non‐parametric continuous data, to determine differences between groups. Relative risks and 95% confidence intervals, comparing the over‐transfused to transfusion required groups, were calculated using binreg (generalised linear models). Statistical significance was specified at *p* < 0.05. Missing data are described, not imputed, and excluded from statistical tests. A small number of patients who did not have a pdHb were excluded from analyses comparing appropriate to over‐transfusion.

## 3 Results

**FIGURE 1:**
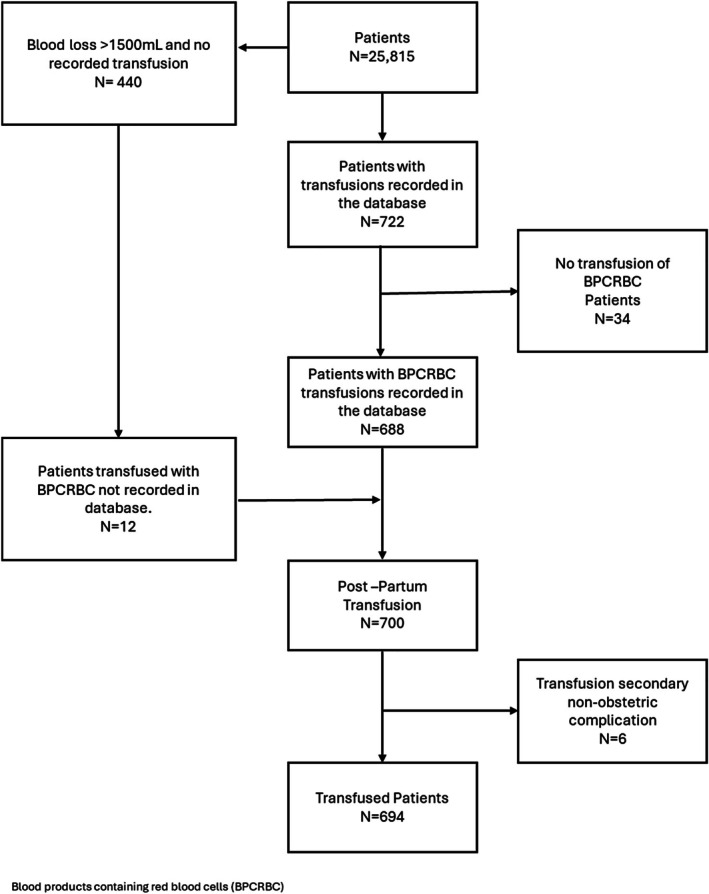
Derivation of transfused cohort.

A total of 26 222 babies were born in 25 815 pregnancies at Te Toka Tumai Auckland Hospital between January 1, 2018, and December 31, 2021. Of the population of 25 815 births, 694 (2.7%) patients were transfused for obstetric indications (Figure 1, Table 1).

**TABLE 1:** Birthing cohort demographic and clinical characteristics and proportion transfused.

		Total		Post‐partum transfused cohort			*p*
		*N* = 25 815		*N* = 694			
		*n*/median	%/IQR	*n*/median	%/IQR	Transfusion %	
Ethnicity (prioritised maternal)	Māori	1924	7.5	64	9.2	3.3	
	Pacific peoples	2898	11.2	128	18.4	4.4	
	Indian	3005	11.6	107	15.4	3.6	
	Other Asian	6406	24.8	204	29.4	3.2	
	Middle Eastern, Latin American, African	1157	4.5	30	4.3	2.6	
	Other European	2963	11.5	50	7.2	1.7	
	New Zealand European	7411	28.7	111	16.0	1.5	< 0.001
	Not stated/missing	51	0.2	0	0.0	0.0	
Mode of birth	Spontaneous vaginal (includes breech)	12 497	48.4	265	38.2	2.1	
	Elective Caesarean Section	5159	20.0	81	11.7	1.6	
	Emergency Caesarean Section	4924	19.1	197	28.4	4.0	
	Forceps	1218	4.7	89	12.8	7.3	
	Ventouse	2017	7.8	62	8.9	3.1	< 0.001
NZ deprivation quintile	1 (least deprived)	4829	18.7	86	12.4	1.8	
	2	5400	20.9	118	17.0	2.2	
	3	5468	21.2	153	22.1	2.8	
	4	4612	17.9	134	19.3	2.9	
	5 (most deprived)	5463	21.2	202	29.1	3.7	< 0.001
	Missing	43	0.2	1			
Plurality	Singleton	25 415	98.5	672	96.8	2.6	
	Multiple	400	1.5	22	3.2	5.5	< 0.001
Maternal age (years)	< 20	312	1.2	13	1.9	4.2	
	20–24	1712	6.6	59	8.5	3.4	
	25–29	5131	19.9	142	20.5	2.8	
	30–34	10 167	39.4	299	43.1	2.9	
	35–39	6872	26.6	144	20.7	2.1	
	≥ 40	1621	6.3	37	5.3	2.3	0.002
Baby birth weight (g)	< 3500	16 131	62.5	435	62.7	2.7	
	3500–4499	9341	36.2	244	35.2	2.6	
	≥ 4500	342	1.3	15	2.2	4.4	0.14
	Missing	1	0.0	0			
BMI (kg/m^2^)	< 18.5	845	3.3	37	5.4	4.4	
	18.5–24.99	13 542	52.5	300	43.5	2.2	
	25–29.99	5921	22.9	170	24.6	2.9	
	30–34.99	2754	10.7	89	12.9	3.2	
	35–39.99	1402	5.4	41	5.9	2.9	
	≥ 40	1098	4.3	53	7.7	4.8	< 0.001
	Missing	253	1.0	4			
Pre‐birth parity	Nulliparous	12 570	48.7	393	56.6	3.1	
	Multiparous	13 245	51.3	301	43.4	2.3	< 0.001
Total blood loss (mL) (median [IQR]) (*n* = 25 796)		400	300, 600	1700	1150, 2400		
	≤ 500	18 276	70.8	41	5.9	0.2	
	501–1500	6694	25.9	256	36.9	3.8	
	1501–2500	678	2.6	263	38.0	38.8	
	> 2500	148	0.6	133	19.2	89.9	< 0.001
	Missing	19	0.1	1			
Gestational age at birth (weeks)	Preterm	2310	8.9	125	18.0	5.4	
	Term	23 505	91.1	569	82.0	2.4	< 0.001

*Note:* Missing data are excluded from statistical testing.

Abbreviation: IQR, interquartile range.

The PPH rate (> 500 mL) of the total cohort was 29.1%. There were differences in the volume of blood loss by ethnicity. Notably, of the most represented ethnic groups (excluding the small number of people with ‘Other’ listed as ethnicity), Pacific people had the highest chance of PPH > 1500 mL at 6.4%, while NZ European people had the lowest chance at 2.3%.

Transfusion was associated with ethnicity and was most frequent among birthing patients of Pacific (4.4%), Indian (3.6%) and Māori (3.3%) ethnicities. The median blood loss of transfused patients was 1700 mL compared to 400 mL in the total cohort. Among transfused patients, 41 (5.9%) were reported as losing ≤ 500 mL. Of patients losing more than 1500 and 2500 mL, 415/811 (51.2%) and 12/145 (8.3%) were not transfused.

Among those transfused, 332/694 (47.8%) were appropriately transfused according to our definition (appropriately transfused), and 325 (46.8%) patients were over‐transfused or had an unnecessary transfusion. Specifically, 136 (19.6%) patients required a transfusion but were over‐transfused and 189 (27.2%) were transfused but did not require any transfusion. There were 37 (5.3%) patients for whom it was not possible to determine if transfusion was necessary as no pdHb was completed. (Table 2).

**TABLE 2:** Demographic and clinical characteristics of transfused patients by appropriateness of transfusion (excludes 37 patients where transfusion requirement unknown).

		Total		Appropriately transfused		Over transfused				RR appropriate compared to over transfusion		
						Transfusion required		Transfusion not required				
		*N* = 657		*N* = 332		*N* = 136		*N* = 189				
		*n*	%	*n*	%	*n*	%	*n*	%	RR	95% CI	*p*
Ethnicity (prioritised maternal)	Māori	62	9.4	35	56.5	13	21.0	14	22.6	1.0		
	Pasifika	116	17.7	78	67.2	15	12.9	23	19.8	1.2	0.9, 1.5	
	Indian	105	16.0	49	46.7	25	23.8	31	29.5	0.8	0.6, 1.1	
	Non‐Indian Asian	191	29.1	88	46.1	39	20.4	64	33.5	0.8	0.6, 1.1	
	MELAA	29	4.4	14	48.3	6	20.7	9	31.0	0.9	0.6, 1.3	
	Other European	49	7.5	20	40.8	13	26.5	16	32.7	0.7	0.5, 1.1	
	NZ European	105	16.0	48	45.7	25	23.8	32	30.5	0.8	0.6, 1.1	0.004
Mode of birth	Spontaneous vaginal birth	249	37.9	151	60.6	44	17.7	54	21.7	1.0		
	Elective caesarean section	77	11.7	31	40.3	22	28.6	24	31.2	0.7	0.5, 0.9	
	Emergency caesarean section	185	28.2	70	37.8	46	24.9	69	37.3	0.6	0.5, 0.8	
	Forceps	85	12.9	40	47.1	17	20.0	28	32.9	0.8	0.6, 1.0	
	Ventouse	61	9.3	40	65.6	7	11.5	14	23.0	1.1	0.9, 1.3	< 0.001
NZ deprivation quintile	1 (least deprived)	80	12.2	36	45.0	15	18.8	29	36.3	1.0		
	2	111	16.9	64	57.7	21	18.9	26	23.4	1.3	0.9, 1.7	
	3	144	21.9	72	50.0	31	21.5	41	28.5	1.1	0.8, 1.5	
	4	130	19.8	68	52.3	28	21.5	34	26.2	1.2	0.9, 1.6	
	5 (most deprived)	191	29.1	92	48.2	41	21.5	58	30.4	1.1	0.8, 1.4	0.4
	Missing data	1	0.2	0	0.0	0	0.0	1	100.0			
Plurality	Singleton	636	96.8	325	51.1	131	20.6	180	28.3	1.0		
	Multiple	21	3.2	7	33.3	5	23.8	9	42.9	0.7	0.4, 1.2	0.2
Maternal age (years)	< 20	12	1.8	6	50.0	2	16.7	4	33.3	0.7	0.4, 1.2	
	20–24	52	7.9	39	75.0	5	9.6	8	15.4	1.0		
	25–29	137	20.9	75	54.7	20	14.6	42	30.7	0.7	0.6, 0.9	
	30–34	285	43.4	132	46.3	66	23.2	87	30.5	0.6	0.5, 0.8	
	35–39	136	20.7	63	46.3	33	24.3	40	29.4	0.6	0.5, 0.8	
	≥ 40	35	5.3	17	48.6	10	28.6	8	22.9	0.6	0.4, 0.9	0.006
Baby birth weight (g)	< 3500	411	62.6	194	47.2	97	23.6	120	29.2	1.0		
	3500–4499	231	35.2	130	56.3	36	15.6	65	28.1	1.2	1.0, 1.4	
	≥ 4500	15	2.3	8	53.3	3	20.0	4	26.7	1.1	0.7, 1.8	0.09
Gestational age at birth (weeks)	Preterm	121	18.4	47	38.8	41	33.9	33	27.3	1.4	1.1, 1.7	
	Term	536	81.6	285	53.2	95	17.7	156	29.1	1.0		0.004
BMI kg/m^2^	< 18.5	34	5.2	14	41.2	7	20.6	13	38.2	1.0	0.6, 1.5	
	18.5–24.99	288	43.8	124	43.1	67	23.3	97	33.7	1.0		
	25–29.99	161	24.5	96	59.6	30	18.6	35	21.7	1.4	1.2, 1.7	
	30–34.99	84	12.8	45	53.6	14	16.7	25	29.8	1.2	1.0, 1.6	
	35–39.99	38	5.8	23	60.5	7	18.4	8	21.1	1.4	1.1, 1.9	
	≥ 40	48	7.3	29	60.4	10	20.8	9	18.8	1.4	1.1, 1.8	0.005
	Missing	4	0.6	1	25.0	1	25.0	2	50.0			
Pre‐birth parity	Nulliparous	369	56.2	190	51.5	70	19.0	109	29.5	1.0		
	Multiparous	288	43.8	142	49.3	66	22.9	80	27.8	1.0	0.8, 1.1	0.6

Abbreviations: CI, confidence interva; MELAA, Middle Eastern Latin American, African; RR, relative risk.

There was no difference in proportion appropriately transfused for any ethnicity group compared to Māori, our pre‐specified referent group. (Table 2). Patients birthed by Caesarean, both elective and emergency, were less likely to be appropriately transfused (RR 0.7 (95% CI 0.5, 0.9) and RR 0.6 (0.5, 0.8) respectively) than people who had spontaneous vaginal births. (Table 2) People < 20 years, and over 25 years, were less likely to be appropriately transfused than people aged 20–24; and people with BMI over 25 more likely to be appropriately transfused compared to people with a normal BMI. (Table 2).

Pre‐delivery haemoglobin, volume of blood loss, and number of units transfused were not associated with appropriate transfusion. Mean pdHb was 83 in the appropriate transfusion group, and 99 in the over‐transfusion group (*p* = 0.0001). (Table 3).

**TABLE 3:** Blood loss and transfusion features of transfused patients by appropriateness of transfusion (excludes 37 patients where transfusion requirement unknown).

		Total		Appropriately transfused		Over transfused				RR appropriate compared to over transfusion		
						Transfusion required		Transfusion not required				
		*N* = 657		*N* = 332		*N* = 136		*N* = 189				
		*n*/mean/median	%/SD/IQR	*n*/mean/median	%/SD/IQR	*n*/mean/median	%/SD/IQR	*n*/mean/median	%/SD/IQR	RR	95% CI	*p*
Total blood loss mL	(Median [IQR]) (*n* = 656)	1798	1180, 2400	1800	1160, 2400	2000	1500, 2950	1500	1000, 2000			0.6
	≤ 500	39	5.9	19	48.7	5	12.8	15	38.5	1.0		
	501–1500	235	35.8	117	49.8	31	13.2	87	37.0	1.0	0.7, 1.4	
	1501–2500	252	38.4	128	50.8	55	21.8	69	27.4	1.0	0.7, 1.5	
	2501–6500	124	18.9	66	53.2	40	32.3	18	14.5	1.1	0.8, 1.6	0.5
	> 6500	6	0.9	1	16.7	5	83.3	0	0.0	0.3	0.06, 2.1	
	Missing	1	0.2	1	100.0		0.0		0.0			
Units RBCs/whole blood transfused	1	294	44.7	146	49.7	0	0.0	148	50.3	1.0		
	2	215	32.7	116	54.0	62	28.8	37	17.2	1.1	0.9, 1.3	
	3	79	12.0	46	58.2	29	36.7	4	5.1	1.2	0.9, 1.5	
	≥ 4	69	10.5	24	34.8	45	65.2	0	0.0	0.7	0.5, 1.0	0.02
Timing of transfusion	Acute (theatre/birthing unit)	366	55.7	172	47.0	102	27.9	92	25.1	0.9	0.7, 1.0	
	Non‐acute (postnatal ward^a^)	290	44.1	160	55.2	34	11.7	96	33.1	1.0		0.07
	Unknown	1	0.2	0	0.0	0	0.0	1	100.0			
Pre‐birth Hb	(mean [SD]) (*n* = 585)	118	16	116	15	118	17	119	17			0.06
Post transfusion Hb	(mean [SD])	91	10	83	4	97	7	100	9			0.0001

Abbreviations: CI, confidence interval; RR, relative risk.

^a^ Includes 3 in intensive care at time of transfusion.

The median number of transfused units was two. Among transfused people, mean pre‐birth haemoglobin was 117 g/L and mean pdHb 90 g/L. (Table 3).

## 4 Discussion

We identified 694 patients who received a transfusion for obstetric indications during the study period. Of the transfused patients, 46.8% either received more units of blood than necessary, or did not require blood transfusion at all. There was no difference in over‐transfusion by volume of blood loss, or between acute and non‐acute settings.

Based on these findings, an anticipated response may be to decrease the use of transfusion because of the previously discussed risks. However, risks associated with under‐transfusion are significant, the most notable of these being maternal death. We did not identify any obstetric haemorrhagic deaths attributed to under‐transfusion in the hospital setting during the study period. Efforts to prevent morbidity and cost from unnecessary blood transfusion must not jeopardise patient safety. This study does not consider under‐transfusion as an outcome and in fact cannot for any outcome except maternal death, an exceedingly rare outcome in New Zealand, due to its retrospective nature. While there are concerns expressed about ability to breastfeed, fatigue and other symptoms in the post‐partum period in people who do not receive a transfusion, these factors are not consistently included in the birth medical record, or indeed in any medical record that our research team can access. The authors recognise that some clinical centres may be tolerant of high rates of over‐transfusion or unnecessary transfusion if it results in low rates of under‐transfusion and its associated complications. Future studies may focus on outcomes in people discharged from hospital with a ‘low’ haemoglobin who do not receive a transfusion.

The proportion of over‐transfusion identified in this study was in line with proportions identified by a previous Australian study of obstetric patients [11]. This team studied 3235 women who delivered in ACT during 2013. Of these 101 received a red cell transfusion and 52% were over‐transfused (with a post transfusion Hb of > 90 g/L) [11]. In contrast the proportion of over‐transfused in the Netherlands was 24%, almost half that of the Australasian studies, despite using more conservative thresholds for over‐transfusion (8.8 and 7.1 g/L for massively bleeding patients and non‐massively bleeding patients respectively) [12]. The ‘4,5,6‐Flexinorm’ transfusion guidelines from the Netherlands were more nuanced than those used in Australasia and appear to result in more desirable transfusion outcomes [12]. Reassessing and refining transfusion guidelines at Te Toka Tumai Auckland Hospital may result in an improvement of transfusion management while providing a safety net to prevent under‐transfusion. Our research team intends to use these individual institution data to develop a predictive model to support transfusion decision‐making in PPH.

We hypothesised that the proportion of over‐transfusion would increase with increased volume of blood loss as when a patient is acutely bleeding clinical teams seek to maintain haemodynamic status without concern about over‐transfusion. However, our data did not reflect this; rate of over‐transfusion did not vary by volume of blood‐loss. This suggests that clinicians prescribing blood products for low volumes of blood loss are equally unconcerned with over‐transfusion as those managing massive bleeding. Alternatively, scenarios which involve high volumes of blood loss often involve more experienced clinicians compared to those with lesser losses being managed by more junior staff.

A hard ‘stop’ (requiring specialist justification) if blood is ordered with a maternal haemoglobin ≥ 80 mg/dL and a requirement for checking of haemoglobin concentration between units of blood given in non‐emergent situations may be simple measures to drive down the rate of over and unnecessary transfusions. Indeed, our finding that 5.3% of patients who received blood did not have a follow‐up haemoglobin indicates that there may not have been significant clinical concern prior to transfusing. Arguably, blood should not have been prescribed in these cases.

Reassuringly transfusion practices at Te Toka Tumai Auckland Hospital appear to not disadvantage Māori and Pasifika patients who have historically had poorer treatment and health outcomes in Aotearoa New Zealand [20]. These two ethnic groups had the highest rates of appropriate transfusions in the study population.

The greatest strength of this study is the size of the data set, including all births that occurred at one of New Zealand's large maternity hospitals. We checked records of all patients identified by the maternity database as transfused and manually reviewed the charts of all patients with > 1500 mL blood loss to confirm we had not omitted any transfusions from the study.

Limitations of the study also include the cohort size as large cohorts can increase the complexity of the data and inevitably result in the possibility of error. Many variables were collected in the hospital electronic record by a large number of individuals with varying levels of clinical experience and knowledge leading to risk of data entry errors. Similarly, the manual collection of data by researchers introduces the potential for further error either in the identification or transcription of data or inadequate documentation; this is a known problem in retrospective studies [21]. Three separate researchers reviewed any discrepancies in transfusion status between digitally collected data and manually checked charts. In addition, the short period of follow up and the narrow area of investigation in this study (whether or not a transfusion was indicated) represent limitations. We did not assess long‐term outcomes for those who received (and did not receive) transfusions as a part of this work. Complications of transfusion such as the formation of maternal antibodies were likewise not collected.

The authors of this study recognise that the drivers of transfusion rates are more complex than haemoglobin values in isolation. Creating decision aid tools to better predict the need for transfusion may increase physical safety for patients and professional safety for healthcare providers, although this needs to be tested in our population. Our group is currently undertaking this work, though we note that there has not been, to date, a highly accurate decision aid created for any population.

In summary, we found that 2.7% of birthing patients received a blood transfusion and approximately half of these patients were over‐transfused or did not require a transfusion at all. Careful action must be taken to prevent under‐transfusion when attempting to reduce over‐transfusion. We appear to be providing transfusions mostly equitably by ethnicity, with encouragingly high rates of Pasifika and Māori women, who receive higher proportions of transfusions, also being appropriately transfused. Predicting the need for transfusion is especially challenging in emergency situations with ongoing bleeding and research on modalities to aid in these decisions are needed. Prevention of unnecessary transfusions on the post‐partum ward, including checking of Haemoglobin after each blood unit is transfused, may provide the most straightforward and safest opportunity to improve care and decrease waste.

## Conflicts of Interest

The authors declare no conflicts of interest.

## Data Availability

The data that support the findings of this study are available from the corresponding author upon reasonable request.
